# A Comparison of Kawasaki Disease during the SARS-CoV-2 Pandemic with Multisystem Inflammatory Syndrome in Children

**DOI:** 10.3390/children11101185

**Published:** 2024-09-28

**Authors:** Tunç Tunçer, Fatih Varol

**Affiliations:** 1 Department of Pediatrics, Division of Pediatric Cardiology, School of Medicine, Bulent Ecevit University, 67000 Zonguldak, Turkey; 2 Department of Pediatrics, Division of Pediatric Intensive Care Unit, Sancaktepe Sehit Prof. Dr. Ilhan Varank Training and Research Hospital, Saglik Bilimleri University, 34785 Istanbul, Turkey; fatih.varol@sbu.edu.tr

**Keywords:** SARS-CoV-2, multisystem inflammatory syndrome in children, Kawasaki disease

## Abstract

Objectives: The purpose of this study was to compare and contrast Kawasaki disease (KD) with multisystem inflammatory syndrome in children (MIS-C) during the SARS-CoV-2 pandemic. Methods: A retrospective analysis of the medical records of patients diagnosed with KD and MIS-C at a single institution from July 2020 to November 2021 was performed. Results: The study included 39 MIS-C patients (84.6% male) with a median age of 138 months and 17 KD patients (58.8% male) with a median age of 36 months. The MIS-C patients were older (*p* < 0.001) and had prolonged hospitalizations (*p* = 0.023), elevated neutrophil counts (*p* < 0.001), C-reactive protein (*p* < 0.001), procalcitonin (*p* < 0.001), interleukin-6 (*p* < 0.014), ferritin (*p* < 0.001), fibrinogen (*p* < 0.001), troponin I (*p* = 0.001), NT-proBNP (*p* < 0.001), and D-dimer levels (*p* < 0.001). There were more cases of hypotension (*p* = 0.024), decreased left ventricular function (*p* = 0.023), and a greater need for corticosteroids (*p* < 0.001), enoxaparin (*p* = 0.045), and therapeutic plasma exchange (*p* < 0.001). Kawasaki disease patients had a greater incidence of rash (*p* < 0.001), changes in oral mucosa (*p* < 0.001), conjunctival injection (*p* < 0.001), extremity changes (*p* < 0.001), and cervical lymphadenopathy (*p* < 0.001). They had a longer duration of fever (*p* < 0.001), elevated white blood cell count (*p* < 0.001), platelet count (*p* < 0.001), and alanine aminotransferase level (*p* < 0.001). The two groups were similar regarding the hemoglobin levels, erythrocyte sedimentation rates, albumin levels, and the frequency of coronary aneurysm, myocarditis, pericarditis, invasive mechanical ventilatory support, and intravenous immunoglobulin treatment. Conclusions: Advanced patient age, a greater presence of gastrointestinal and cardiac findings associated with hypotension, increased NT-proBNP levels, decreased left ventricular function, the use of various treatment modalities, and longer hospital stays suggest MIS-C, whereas prolonged fever and classical clinical features of KD favor KD.

## 1. Introduction

Subsequent to the onset of the COVID-19 health crisis, an inflammatory, multisystem illness in children was identified in Europe and the USA, exhibiting clinical symptoms akin to those of Kawasaki disease (KD), emerging in the weeks after SARS-CoV-2 infection [1,2,3,4,5,6,7]. Case criteria for this entity were established by the Centers for Disease Control and Prevention (CDC) and the World Health Organization (WHO) [8,9]. This condition has been described using a number of other terms; however, for the purposes of this paper, the acronym MIS-C will be used. The diagnosis of MIS-C requires the following criteria as per the CDC definitions: fever, involvement of at least two systems or organs, elevated inflammatory markers, previous exposure or infection with SARS-CoV-2, and ruling out other possible causes [9] (Table 1).

Kawasaki disease (KD) is characterized by a sudden onset of fever and an unexplained cause; most patients with this illness are under the age of five [10]. The diagnostic method for KD is clinical criteria. In order to diagnose Kawasaki disease, a patient must have a fever that lasts five days or more in addition to four out of five of the following symptoms: bilateral conjunctival hyperemia, alterations in the lips and oral cavity, lymphadenopathy in the cervical region, redness of the hands and feet, swelling during the acute phase, and/or desquamation around a fingernail or toenail in the subacute phase, and polymorphous rash. [10]. If a child has a fever lasting five days or longer in addition to two or three of these symptoms, their condition is classified as incomplete (atypical) KD [10] (Table 1). Due to mucocutaneous symptoms, MIS-C was initially referred to as a “Kawasaki-like” disease [2,4]. Both KD and MIS-C share a number of common clinical features. These include erythema with edema of the extremities, fever, rash, conjunctivitis, and cervical lymphadenopathy. Given some clinical similarities, KD and MIS-C may belong to the same group of inflammatory illnesses [11]. There are, however, clinical characteristics where KD and MIS-C diverge. MIS-C causes more gastrointestinal problems, shock, and coagulopathy, while these are of lesser frequency in the KD [12]. Myocardial inflammation, coronary artery aneurysms, and systolic and diastolic dysfunction of the left ventricle are frequent signs of substantial cardiac involvement in patients with MIS-C [11]. MIS-C is more prevalent among individuals of Hispanic, African, or Latino descent, whereas classic KD is more frequently observed in Northeast Asian countries [4,12,13,14,15]. Children under five are more likely to experience KD, but older children are more likely to experience MIS-C [12]. The recognized distinctions in epidemiology, pathology, inflammation, and immunity between KD and MIS-C indicate that these conditions are separate entities [1,2,3,4,5,6,7,11,16]. Pediatricians have encountered difficulties in distinguishing between patients presenting with MIS-C and those exhibiting symptoms of KD during the SARS-CoV-2 pandemic [17,18]. Although numerous studies compared pre-pandemic KD with MIS-C, few studies investigated the differences between pandemic KD and MIS-C [2,10,19,20,21,22,23,24]. This study aims to identify the unique features facilitating differentiation between pandemic KD and MIS-C.

## 2. Materials and Methods

### 2.1. Study Population and Protocol

The Sancaktepe Şehit Prof. Dr. İlhan Varank Training and Research Hospital Scientific Research Ethics Committee gave its clearance for the study’s conduct (Date: 10 February 2021, Decision No: 2021/125). All methods followed the ethical guidelines and principles of the Declaration of Helsinki. Patient consent was exempted due to the retrospective nature of our investigation.

Patients hospitalized with KD and MIS-C between July 2020 and November 2021 were the subjects of this retrospective single-center study at the Departments of Pediatrics of Sancaktepe Şehit Prof. Dr. İlhan Varank Training and Research Hospital. This study included 17 KD patients admitted to the pediatric cardiology outpatient clinic and 39 MIS-C patients in intensive care and pediatric inpatient clinics. While all KD patients fulfilled the American Heart Association criterion, all MIS-C patients satisfied the CDC criteria [9,10] (Table 1). The hospital database provided information on the demographics, clinical characteristics, laboratory findings, echocardiographic findings at admission, duration of fever, treatments, and length of stay of patients. All of these features were examined in all patients of the study.

Laboratory findings upon admission included C-reactive protein (CRP), white blood count (WBC), procalcitonin, neutrophil percentage, hemoglobin (Hgb), platelet count, erythrocyte sedimentation rate (ESR), interleukin-6 (IL-6), alanine aminotransferase (ALT), ferritin, troponin, D-dimer, N-terminal prohormone of brain natriuretic peptide (NT-proBNP), and fibrinogen. Patients were tested for COVID-19 by evaluating the IgM and IgG levels of antibodies, as well as COVID-19 PCR results. Cultures of blood, urine, and cerebrospinal fluid (if a central nervous system infection was suspected), along with viral serology and a respiratory pathogen PCR panel, were utilized to identify or rule out other potential causes. The echocardiographic findings examined were as follows: coronary aneurysm, left ventricular function, myocarditis, and pericarditis. Treatments included 2 g/kg intravenous infusion of immunoglobulin (IVIG), 1–4 mg/kg of intravenous methylprednisolone, 1 mg/kg of subcutaneous enoxaparin, anakinra 2–4 mg/kg of subcutaneous anakinra, therapeutic plasma exchange, and invasive mechanical ventilatory support.

### 2.2. Echocardiographic Assessment

Using the Philips Affiniti 50 c, transthoracic echocardiography was conducted (Philips Healthcare, Andover, MA, USA). The same pediatric cardiologist evaluated all subjects. The echocardiographic modes utilized were the M-mode, 2D, color, pulsed, and continuous-wave Doppler. The absolute diameters (in millimeters) and z-scores of the proximal coronary arteries, which included the left main coronary artery, left anterior descending coronary artery, left circumflex coronary artery, and right coronary artery, were measured. Fractional shortening of less than 28% was considered to indicate reduced left ventricular systolic performance. In accordance with the Kawasaki recommendations established by the American Heart Association, z-scores were used to describe coronary aneurysms. Small aneurysms were characterized by z-scores ranging from 2.5 to 5.0, medium aneurysms by z-scores between 5 and 10 with an absolute diameter of less than 8 mm, and large aneurysms by z-scores between 10 and 8 mm [10].

### 2.3. Statistics

We used Statistical Package for Social Science Statistics software, version 17.0 (IBM Corp., Armonk, NY, USA) for the statistical analysis. Normality was evaluated using the Kolmogorov–Smirnov test and histograms. The data are presented as the frequency, percentage, median, mean, standard deviation, minimum, maximum, and interquartile range (IQR). We used Fisher’s exact test or Pearson’s chi-squared test to compare the categorical variables. Using the Mann–Whitney U test, continuous variables without a normal distribution were compared, and *p*-values of less than 0.05 were considered statistically significant. The effect size was determined to be d: 1.185 in the power analysis performed with the G Power 3.1.9.7 software (Franz Faul, Kiel, Germany). Based on the established effect size and a 5% error margin, the study’s power was assessed to be 97.96%.

## 3. Results

This study included 39 children with MIS-C and 17 children with KD. At the time of diagnosis, the median ages of the patients with KD and MIS-C were 36 (18–48) and 138 (7–214) months, respectively. MIS-C patients exhibited a higher age compared to KD patients (*p* < 0.001). Twenty-three MIS-C patients (58.9%) and ten KD patients (58.8%) were male, and the groups did not differ statistically based on sex (*p* = 0.992). Table 2 delineates the characteristics of all participants in the study.

Of the 33 patients diagnosed with MIS-C, 34 (84.6%) tested positive for SARS-CoV-2 serology, and 15 (1.5%) tested positive for PCR; furthermore, 29 MIS-C patients (74.3%) reported being exposed to SARS-CoV-2. The COVID-19 vaccination had been administered to four patients. All patients were free of earlier SARS-CoV-2 infections. The mean time between COVID-19 onset and MIS-C development was 18.1 ± 4 days. No COVID-19 or SARS-CoV-2 exposure history was present in any of the KD patients. Both the serology and PCR assays for SARS-CoV-2 were negative in these individuals. At least two of the five main clinical characteristics of KD—rash, changes in the oral mucosa, bilateral nonexudative conjunctivitis, cervical lymphadenopathy, and hand and foot erythema and edema—were present in eight patients (20.5%) with MIS-C. Three (17.6%) of the seventeen patients with Kawasaki disease were diagnosed with atypical Kawasaki disease.

The frequency of rash (*p* < 0.001), changes in mouth mucosa (*p* < 0.001), lymphadenopathy in the cervical region (*p* < 0.001), bilateral conjunctival injection (*p* < 0.001), and hand and foot erythema and edema (*p* < 0.001) were more pronounced in patients with Kawasaki disease (KD). In contrast, the incidence of acute gastrointestinal signs (*p* < 0.001), acute respiratory manifestations (*p* = 0.001), and hypotension (*p* = 0.024) was elevated in patients with MIS-C (Table 2). Higher neutrophil percentages were seen in MIS-C patients (*p* < 0.001), as well as higher CRP (*p* < 0.001), procalcitonin (*p* < 0.001), ferritin (*p* < 0.001), fibrinogen (*p* < 0.001), D-dimer (*p* < 0.001), IL-6 (*p* = 0.014), troponin I (*p* = 0.001), and BNP values (*p* < 0.001). On the other hand, patients with KD exhibited elevated ALT values (*p* < 0.001), increased white blood cell counts (*p* < 0.001), higher platelet counts (*p* < 0.001), and prolonged fever duration (*p* < 0.001). No statistical difference was observed in the Hgb, ESR, and albumin levels among the two patient cohorts (Table 3).

The echocardiography results indicated no statistically significant differences between the two patient groups regarding myocarditis, pericarditis, or coronary artery disease (*p* = 0.309, *p* = 0.088, and *p* = 0.546) (Table 4). However, patients with MIS-C exhibited a higher incidence of diminished left ventricular function (*p* = 0.023) (Table 4).

The treatments applied and the number of subjects that received treatments are depicted in Table 4. None of the Kawasaki patients required intensive care, although 35 (89.7%) of the MIS-C patients did. Of the patients needing intensive care, 22 (62.8%) received therapeutic plasma exchange treatment. Three patients with MIS-C (7.6%) were placed on extracorporeal membrane oxygenation. IVIG was administered to all KD patients, whereas 84.6% of the MIS-C group received it. Patients with KD did not receive intravenous corticosteroids, subcutaneous enoxaparin, anakinra, invasive mechanical ventilatory support, or therapeutic plasma exchange treatments. More patients with MIS-C received intravenous corticosteroids (*p* < 0.001), subcutaneous enoxaparin (*p* = 0.045), and therapeutic plasma exchange (*p* < 0.001). The two patient cohorts exhibited no statistically significant differences regarding IVIG, anakinra therapy, and invasive mechanical ventilation support (Table 4).

MIS-C patients were in the hospital longer than the KD patients (*p* = 0.023), although the KD patients had a statistically increased number of days with fever (*p* < 0.001) (Table 2 and Table 4).

## 4. Discussion

Numerous researchers have examined the distinctions between classical KD and MIS-C, comparing MIS-C patients with pre-pandemic KD patients as well as pre-pandemic KD patients with pandemic KD patients [2,12,24,25,26]. Nevertheless, the quantity of comparative studies of MIS-C and pandemic KD is limited, including the studies that compare MIS-C with pre-pandemic plus pandemic KD [27,28,29,30,31]. The findings from our study provide insights into the comparative features of pandemic KD and MIS-C, particularly in light of the COVID-19 epidemic. Both conditions share overlapping clinical features but exhibit distinct differences that are crucial for accurate diagnosis and management.

The patient’s age may be useful in differentiating between the two conditions. The demographic differences observed in our research of MIS-C patients—namely that they were older—support previous reports [11,12,23,24]. These results align with previous research that compared pandemic patients with KD to MIS-C patients [27,30,31].

Our study supports the existing knowledge about the differences in clinical features between pre-pandemic KD and MIS-C. It indicates that conjunctivitis, rash, oral mucosal changes, and changes in extremities occur more frequently in KD patients compared to MIS-C patients [19,23,24]. Conversely, patients with MIS-C are more likely to experience gastrointestinal symptoms, acute respiratory difficulties, and low blood pressure [19,24]. Additionally, KD patients’ fevers last longer [19,23,24]. Nevertheless, MIS-C and KD may share certain clinical characteristics. According to the Phi et al. study, individuals who have MIS-C had a considerably increased prevalence of gastrointestinal symptoms, hand and foot edema or erythema, and generalized skin rash [20]. Patients did not differ in terms of conjunctivitis and skin rash in the research conducted by Yavuz et al. [23]. They also found that oropharyngeal signs, cheilitis, edema of the extremities, and lymphadenopathy in the cervical region were more common in patients with Kawasaki disease [23]. When considering the studies comparing patients with KD and those with MIS-C during the pandemic, fever was more prolonged in KD patients, which is in line with our study [27,31]. Consistent with similar studies, hypotension and acute gastrointestinal issues were more common among MIS-C patients [27,29,31]. However, even though the frequency of the clinical features of changes in the oral mucosa, conjunctivitis, rash, cervical lymphadenopathy, and redness and edema of extremities was higher in patients with KD in our study, in the study by Şener et al., conjunctivitis and changes in the extremities did not differ between the groups [27]. Only 20.5% of the MIS-C patients in our study exhibited at least two significant clinical features of KD. However, all the patients with MIS-C had Kawasaki disease-like symptoms in the study by Şener et al., which may account for the discrepancy between the two studies’ findings. Moreover, a study by Alkan et al., using a methodology similar to ours, did not reveal a significant difference regarding rash and conjunctivitis between the two groups [31]. These results might validate the clinical feature overlap between the two diseases.

According to our research, MIS-C patients had reduced WBC and platelet counts alongside increased neutrophil, CRP, procalcitonin, IL-6 ferritin, fibrinogen, and D-dimer levels, compared to KD patients, indicating hyperinflammation and cardiovascular involvement. Our data were largely consistent with most studies in the literature [1,2,12,21,27,29,31,32]. However, there are also contradicting results in the literature comparing pre-pandemic KD patients with MIS-C patients. Ferritin, CRP, ESR, and D-dimer levels did not significantly differ between the groups in the Cem et al. study, nor did the WBC, platelet, ALT, and CRP values in the study by Phi et al. [20,24]. In addition, Yavuz et al. found no significant differences in the CRP, ALT, fibrinogen, or D-dimer levels between the two groups; in fact, their investigation found that the average ESR level of the Kawasaki disease group was significantly greater [23]. We interpreted the studies comparing pandemic KD patients and MIS-C patients in terms of laboratory data. Şener et al. found no significant difference in ALT or ESR between the groups, which is consistent with our findings. In contrast, Mehrban et al. found higher fibrinogen and ESR levels in KD patients, with no statistical difference between the groups regarding the D-dimer and CRP levels [27,28,29,30]. Discrepancies regarding the laboratory data among various studies may be related to the different geographies and ethnicities where the studies were conducted. Kawasaki disease prevalence is higher among the Asian countries of Japan, South Korea, China, and Taiwan than in the USA and Europe [10]. Children of Hispanic or Black origin had the highest prevalence of MIS-C, whereas Asian children had the lowest incidence [11]. While there was no difference in the CRP, ferritin, fibrinogen, and D-dimer levels across the groups in the aforementioned studies, our study found statistical differences regarding these data between the two groups. The fact that a majority of our MIS-C patients (89.7%) required intensive care unit therapy may be the reason behind this. Of these, 62.8% were severe enough to receive therapeutic plasma exchange treatment, and three MIS-C patients were placed on extracorporeal membrane oxygenation as well, which elevated these serum markers, leading to a more pronounced systemic inflammatory reaction in MIS-C compared to KD. Nonetheless, we think more investigation is necessary to ascertain the optimal cut-off values for these laboratory data, allowing their use as markers for the comparison of both diseases, as there have been many conflicting results.

Cardiovascular complications are the most significant manifestation of MIS-C. Consistent with both studies comparing pre-pandemic or pandemic KD patients with MIS-C patients, the echocardiograms of MIS-C individuals showed a greater frequency of decreased left ventricular systolic function compared to the KD patients [7,23,27,30,31,33]. The two groups did not differ in terms of the frequencies of coronary aneurysms in our study. When the pre-pandemic KD patients were compared with the MIS-C patients, in the Phi et al. study, aneurysm or coronary artery dilatation was more commonly seen in MIS-C [20]. Additionally, Yavuz et al. demonstrated that the MIS-C group’s coronary diameter was greater than that of the KD group, as it was greater than 2.5 mm in 71% of MIS-C patients [23]. Contrary to these findings, Cattalini et al. and Matsubara et al. indicated that, compared to KD patients, there are fewer coronary artery abnormalities in MIS-C, and those that do exist are almost invariably temporary and recover quickly over time [12,21,33]. In a cohort of 1.733 people in the US, 16.5% of patients with MIS-C developed coronary artery anomalies, a percentage similar to those with untreated KD [34]. In line with these studies, Cem et al.‘s study indicated that KD patients had a statistically greater coronary dilatation rate on echocardiography [24]. Data obtained from studies conducted during the pandemic were consistent with our study. A single research study found that MIS-C patients had a lower incidence of coronary artery dilatation, and the coronary artery z-scores of the two groups did not significantly differ according to two other studies [27,31,35]. The difference in the coronary dilation rates between the KD patients before the pandemic and patients during the pandemic may be explained by the early admission of KD patients during the pandemic, owing to concerns about contracting COVID-19. This heightened awareness of Kawasaki-like disease among physicians likely led to the prompt recognition and treatment of the condition so that patients often received intravenous immunoglobulin (IVIG) treatment within a week of developing a fever [36].

Our study indicated elevated levels of troponin and NT-proBNP alongside a higher incidence of hypotension in MIS-C. The two groups did not significantly differ in terms of myocarditis or pericarditis. Research has revealed that symptomatic myocarditis affects 40–80% of MIS-C patients [2,6,7,37]. However, studies comparing pre-pandemic KD patients with MIS-C patients regarding these data have obtained conflicting results. In terms of myocarditis or pericarditis, Phi et al.‘s investigation did not find any significant differences, whereas research by Cem et al. and Yavuz et al. showed that there was a higher incidence of pericarditis and pericardial effusion in MIS-C [20,23,24]. Myocarditis and pericarditis did not significantly differ between the groups during the pandemic in one research study [27]. Conversely, another study indicated that pericardial effusion was significantly greater in the MIS-C group [31]. Cardiac biomarkers, including troponin and NT-pro-BNP levels, are much higher in MIS-C compared to previous KD cohorts, indicating cardiac insufficiency and myocardial damage resulting in hypotension [12]. Studies comparing MIS-C to pre-pandemic and pandemic KD revealed that MIS-C patients had significantly raised cardiac troponin and NT-pro-BNP levels, as well as an increased frequency of hypotension [1,7,12,21,24,27,29,31,32,35,37]. This is consistent with our findings, although not all data points were examined in every study.

The treatment modalities employed also differed between the two groups in our study. All KD patients received IVIG, which is a standard treatment, compared to 84.6% of the MIS-C group. The MIS-C group more frequently received intravenous corticosteroids, subcutaneous enoxaparin, and therapeutic plasma exchange. However, there was no statistical difference in the frequency of IVIG, anakinra, and invasive mechanical ventilatory support treatments. Three patients with MIS-C (13.6%) also required extracorporeal membrane oxygenation. IVIG and low-molecular-weight heparin use did not significantly differ between pre-pandemic Kawasaki disease and MIS-C patients, according to a study [31]. The rate of steroid use was substantially greater in MIS-C [31]. The KD patients did not require inotropic support or intensive care [31]. On the contrary, the study by Ciftdogan et al., conducted during the pandemic, found that patients with clinical characteristics similar to KD were treated more frequently with IVIG [29]. The study conducted by Şener et al. during the pandemic found that, in the MIS-C group, intravenous corticosteroids, anakinra, and the use of therapeutic plasma exchange were much greater [27]. The use of different treatment methods in the studies could be due to the involvement of different patient groups. Pre-pandemic KD patients, pandemic KD patients, and MIS-C patients with or without features overlapping with KD and different treatment needs may account for the various treatment methods. These data reflect the more severe and complex nature of MIS-C, which often requires a multi-modal and patient-specific approach to manage the hyperinflammation and organ dysfunction associated with the syndrome [17]. In contrast, KD patients were primarily treated with IVIG, consistent with established guidelines [10].

One of the most striking differences was the duration of hospitalization in our study. Although KD patients experienced a longer duration of fever, MIS-C patients had a longer overall hospital stay. This could be attributed to the more severe and multisystemic nature of MIS-C, which may require prolonged treatment and monitoring. This fact is also supported by studies conducted with pre-pandemic or pandemic KD patients vs. patients with MIS-C [23,27,35]. However, a study conducted by Yavuz et al. revealed no significant difference in hospitalization length between the groups [23].

A summary of the literature findings between MIS-C and Kawasaki disease is depicted in Table 5.

## 5. Conclusions

In summary, while KD and MIS-C share several clinical features, our study underscores the importance of recognizing the distinct characteristics of pandemic KD and MIS-C in particular. Advanced patient age, an increased presence of gastrointestinal and cardiac findings associated with hypotension, increased NT-proBNP levels, and decreased left ventricular function, as well as various treatment modalities and a longer hospital stay suggest MIS-C, whereas a longer fever duration and the classical clinical features of KD favor KD. Future research should continue to explore these differences and improve the diagnostic criteria to enhance patient outcomes.

## 6. Limitations

The principal limitation of our study is the limited sample size of patients in the KD group. The study’s second issue is that not all of our KD patients shared the overlapping features of KD. These two restrictions may be attributed to the significant decrease in the number of patients with classic KD during the pandemic [38]. Most patients with MIS-C in this study required intensive care, receiving treatments such as ECMO and plasma exchange. Baseline differences in severity between the two cohorts (intensive care unit admission rates: 89.7% vs. 0%) account for potential bias in their comparisons.

## Figures and Tables

**Table 1:** Diagnostic criteria for MIS-C, classical and atypical KD.

MIS-C	A person under 21 years who exhibits a temperature of 38.0 °C or higher for at least 24 h, or a report of a 24 h or longer subjective fever and signs of inflammation, including but not restricted to elevated levels of ESR, CRP, procalcitonin, ferritin, fibrinogen, lactic acid dehydrogenase, D-dimer or interleukin-6, as well as decreased lymphocytes, low albumin, and increased neutrophils, with clinically severe disease necessitating hospitalization, and multisystem involvement with two or more of dermatologic, renal, gastrointestinal, respiratory, hematologic, cardiac or neurological organs; AND No viable alternative diagnoses; AND Exposure to COVID-19 within the four weeks preceding symptom onset or a positive result for recent or active SARS-CoV-2 infection, as confirmed by antigen testing, serology, or RT-PCR. Extra remarks If a person meets the MIS-C case definition, they still need to be reported, regardless of whether they, in whole or in part, satisfy the criteria for Kawasaki disease. All cases of juvenile death associated with SARS-CoV-2 infection must be assessed for MIS-C.
Classical KD	When a patient has a fever for a minimum of five days (the onset of fever is regarded as the initial day of the fever) and at least four of the five main clinical characteristics listed below, they are diagnosed with classic KD. The diagnosis of Kawasaki disease (KD) can be established after 4 days of fever, provided that at least four principal clinical features are present, especially when there is swelling and redness in the feet and hands. However, in rare cases, after three days of fever, doctors who have treated numerous KD patients may diagnose the patient. Typically unilateral, with a minimum diameter of 1.5 cm lymphadenopathy in the cervical region; Redness and cracking of the lips, tongue that looks like a strawberry, and/or redness of the mouth and pharyngeal mucosa; Injections of both bulbar conjunctiva without exudate on both sides; Hand and foot erythema and edema in the acute phase and/or desquamation around a fingernail or toenail in the subacute phase; Rash patterns that resemble erythema multiforme, maculopapular rash, or diffuse erythroderma.
Atypical KD	Any infant or child with a fever that will not go away, with fewer than four of the main symptoms and relevant laboratory or echocardiographic findings, should undergo evaluation for the diagnosis of incomplete (or atypical) Kawasaki disease. Laboratory tests are performed for infants presenting with idiopathic fever for at least 7 days or a fever lasting for at least 5 days and satisfying two or three diagnostic criteria. ➢ When the ESR is less than 40 mm/h, low CRP levels (<3 mg/dL), and the fever continues, serial clinical and laboratory examinations are performed. Echocardiography is performed when peeling starts. ➢ The treatment is initiated if a CRP level of 3 mg/dL or more and an ESR of 40 mm/h or more are detected with three or more of the laboratory findings below or with a positive echocardiogram. Anemia for age; Albumin level less than 3 g/dL; A high ALT level; After the seventh day of fever, the platelet count should be 450,000/mm^3^; White blood cell count exceeding 15,000 mm^3^; If there are more than ten WBCs per high-power field.

Abbreviations: ESR—erythrocyte sedimentation rate; CRP—C-reactive protein; RT-PCR—reverse transcription-polymerase chain reaction; ALT—alanine aminotransferase; WBC—white blood cell.

**Table 2:** Characteristics, symptoms, and signs of patients.

	MIS-C Patients (*n* = 39)	KD Patients (*n* = 17)	*p*
Age (months)			<0.001
Median (min–max)	138 (7–214)	36 (18–48)	
IQR (*p* 25–*p* 75)	(97–182)	(32–42)	
Sex, *n* (%)			0.992
Male	33 (84.6)	10 (58.8)	
Female	6 (15.3)	7 (41.1)	
Clinical symptoms			
Days of fever			<0.001
Median (min–max)	3 (1–7)	6 (5–7)	
IQR (*p* 25–*p* 75)	(3–5)	(5–6)	
Rash, *n* (%)	2 (5.1)	12 (70.5)	<0.001
Oral mucosal changes, *n* (%)	4 (10.2)	14 (82.3)	<0.001
Bilateral conjunctival injection, *n* (%)	6 (15.3)	12 (70.5)	<0.001
Erythema and edema of extremities, *n* (%)	0 (0)	6 (35.2)	<0.001
Cervical lymphadenopathy, *n* (%)	6 (15.3)	15 (88.2)	<0.001
Acute gastrointestinal symptoms, *n* (%)	22 (66.6)	2 (11.7)	<0.001
Acute respiratory symptoms, *n* (%)	17 (43.5)	0 (0)	0.001
Hypotension, *n* (%)	11 (28.2)	0 (0)	0.024

**Table 3:** Laboratory results of the patients.

Laboratory Results Median (Min–Max) IQR (*p* 25-*p* 75)	MIS-C Patients	KD Patients	*p*
White blood cell count (×10^3^/μL)	7600 (500–31,000) (4800–11,300)	15,000 (12,200–17,600) (14,400–16,000)	<0.001
Hemoglobin, g/dL	10.4 (7–15.4) (9.3–12.9)	11 (10–12) (10.5–11.3)	0.568
Neutrophil (%)	85 (26–95) (75–90)	57 (43–75) (49–65)	<0.001
Platelet count (×10^3^/uL)	180 (7–446) (99,000–263,000)	290 (220–400) (270,000–360,000)	<0.001
C-reactive protein, mg/dL (<0.5)	113 (1.5–329) (18–190)	14 (9–35) (12–17)	<0.001
Erythrocyte sedimentation rate, mm/h (<20)	34 (4–114) (23–68)	40 (24–48) (35–43)	0.859
Procalcitonin ng/mL (<0.05)	3.18 (0.01–168) (0.47–16)	0.02 (0.01–0.05) (0.01–0.03)	<0.001
Interleukin-6, pg/mL (<5.9)	100 (2.48–2000) (12–280)	28 (6–45) (14–38)	0.014
Alanine aminotransferase, IU/L (<39)	22 (5–381) (14–38)	60 (40–84) (55–67)	<0.001
Albumin g/L (38–54)	31 (11.1–48) (28–39)	30 (20–42) (24–35)	0.208
Ferritin, ng/mL (<207)	454 (27–3381) (198–1143)	80 (20–145) (45–120)	<0.001
Fibrinogen mg/L (<400)	485 (176–724) (408–585)	280 (210–550) (230–400)	<0.001
D-Dimer, mg/L (<0.5)	3.4 (0.34–35) (1.8–5.19)	0.8 (0.2–1.4) (0.5–1)	<0.001
Troponin I, picogram/mL (<0.14)	60 (0–106,695) (22–146)	10 (3–18) (6–12)	0.001
N-terminal pro-brain natriuretic peptide pg/mL (<100)	980 (10–35,000) (300–2010)	45 (12–90) (34–75)	<0.001

**Table 4:** Echocardiographic findings, treatments, and hospitalization duration of patients.

Echocardiographic Findings, *n* (%)	MIS-C Patients	KD Patients	*p*
Coronary aneurysm	5 (12.8)	0 (0)	0.309
Decreased left ventricular function	10(25.6)	0 (0)	0.023
Myocarditis	7 (17.9)	0 (0)	0.088
Pericarditis	3 (7.69)	0 (0)	0.546
Treatments, *n* (%)			
Intravenous immunoglobulin	33 (84.6)	17 (100)	0.087
Corticosteroids	30 (76.9)	0 (0)	<0.001
Subcutaneous enoxaparin	9 (23)	0 (0)	0.045
Anakinra	3 (7.69)	0 (0)	0.546
Therapeutic plasma exchange	22 (56.4)	0 (0)	<0.001
Invasive mechanical ventilatory support	7 (17.9)	0 (0)	0.088
Hospitalization duration, days, median (min–max)	6 (1–12)	4 (2–6)	0.023

**Table 5:** Summary of the literature findings between MIS-C and Kawasaki disease.

	MIS-C	Kawasaki Disease
Age	Older	Young
Days of fever		
Clinical features (rash, conjunctivitis, etc.)	Less common	More common
Acute gastrointestinal symptoms	More common	Less common
Acute respiratory symptoms	More common	Less common
Hypotension	More common	Less common
Laboratory results	Increased neutrophil, CRP, procalcitonin, IL-6 ferritin, fibrinogen, D-dimer, troponin, NT-pro-BNP Reduced WBC and platelets	Increased ESR and platelets
Echocardiographic findings	Decreased left ventricular systolic function Coronary artery abnormalities (comparative frequency is debatable) Myocarditis	Coronary artery abnormalities (comparative frequency is debatable)
Treatments	IVIG Intravenous corticosteroids Subcutaneous enoxaparin Therapeutic plasma exchange Invasive mechanical ventilatory support	IVIG
Hospitalization duration	Long	Short

Abbreviations: ESR—erythrocyte sedimentation rate; CRP—C-reactive protein; WBC—white blood cell; IVIG—intravenous immunoglobin.

## Data Availability

The original contributions presented in the study are included in the article, further inquiries can be directed to the corresponding author.
